# Meta-analysis of the safety and efficacy between pulsed field ablation vs. traditional ablation for persistent atrial fibrillation

**DOI:** 10.3389/fcvm.2026.1839858

**Published:** 2026-08-03

**Authors:** Hui Li, Qin Cui, Weiping Tao, Qinghua Qiu, Aiwu Guo

**Affiliations:** Department of Cardiology, Nantong Rici Hospital, Yangzhou University, Nantong, China

**Keywords:** meta-analysis, persistent atrial fibrillation, pulsed field ablation, safety and efficacy, traditional ablation

## Abstract

**Background:**

Pulsed field ablation (PFA) has emerged as an innovative non-thermal approach with significant promise for treating atrial fibrillation (AF). We performed a meta-analysis between PFA and traditional ablation (TA, including cryoballoon ablation [CBA] and radiofrequency ablation [RFA]) for persistent atrial fibrillation (PerAF).

**Methods:**

A systematic search was conducted in the Medline, PubMed, Embase, and Cochrane Library databases to include all relevant studies that compared PFA with TA.

**Results:**

Six trials involving 1,610 patients were included in the study. Pooled analyses revealed that PFA exerted significant advantages over TA, characterized by lower recurrence of atrial tachyarrhythmias (ATAs) (risk ratio [RR]: 0.83; 95% CI: 0.70 to 0.97; *P* = 0.02). Further analysis revealed no significant difference when PFA was compared with CBA alone (RR: 0.89, 95% CI: 0.74 to 1.08, *P* = 0.24), whereas PFA was superior to RFA (RR: 0.75, 95% CI: 0.59 to 0.96, *P* = 0.02). For AF recurrence, PFA also demonstrated a lower rate compared with TA (RR: 0.79, 95% CI: 0.65 to 0.95, *P* = 0.01), and this advantage was also observed in the comparison with CBA (RR: 0.71, 95% CI: 0.52 to 0.99, *P* = 0.04). No significant difference was found between PFA and TA in terms of the atrial tachycardia/atrial flutter (AT/AFL). In addition, PFA significantly shortened the procedural time (standard mean difference [SMD]: −1.25, 95% CI: −1.87 to −0.64, *P* < 0.00001) and left atrial dwell (LAD) time (SMD: −0.51, 95% CI: −0.99 to −0.03, *P* = 0.04). However, PFA required a longer fluoroscopy time compared with RFA (SMD: 1.67, 95% CI: 1.12 to 2.22, *P* < 0.00001). There was no significant difference in the incidence of complications between the two groups.

**Conclusion:**

PFA is effective and safe for PerAF treatment, featuring shorter procedure time and lower ATAs recurrence.

## 1 Introduction

Atrial fibrillation (AF) stands as the most frequently encountered sustained cardiac arrhythmia (1). Over the past decade, with advances in technology and the accumulation of operators' experience, catheter ablation (CA) has become a first-line treatment for AF (2). Pulmonary vein isolation (PVI)—the foundational intervention for AF, is primarily performed using traditional ablation (TA) techniques such as cryoballoon ablation (CBA) or radiofrequency ablation (RFA) (3, 4). Although TA effectively achieves PVI, its non-specific tissue damage can damage adjacent tissues, potentially giving rise to complications like phrenic nerve injury (PNI), PV stenosis, or the most critical, an atrio-esophageal fistula (5, 6).

A novel non-thermal technology known as pulsed field ablation (PFA) has recently emerged (7). PFA provides multiple advantages, including tissue-selective injury, speed, and a promising long-term durability profile (8, 9). By delivering high-intensity electric fields, PFA triggers targeted tissue necrosis while minimizing collateral damage to adjacent structures (10). Several studies have shown that PFA achieves PVI with both high success rate and low complication rate (11–13). However, for persistent atrial fibrillation (PerAF), data on whether PFA also offers high success and safety are limited and the results are inconsistent. Therefore, we conducted an meta-analysis aimed at evaluating the clinical benefits of PFA in PerAF.

## 2 Methods

**Figure 1:**
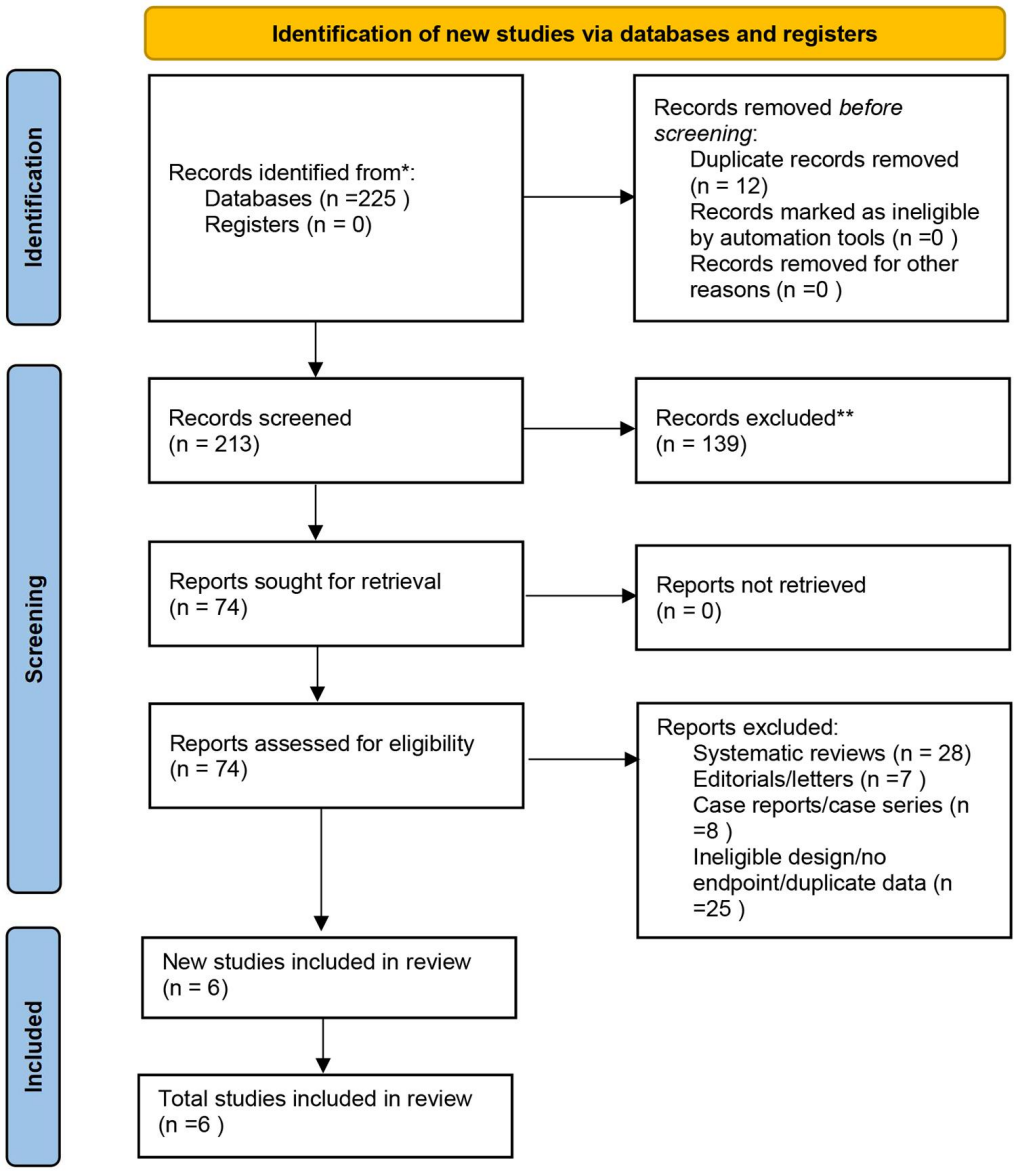
PRISMA flowchart of the screening process.

This study was registered prospectively at INPLASY (Registration No. NPLASY202560081) and was performed in accordance with the PRISMA 2020 guidelines. A complete PRISMA checklist is provided as Supplementary Material. The detailed study selection process is presented in the PRISMA flow diagram (Figure 1), with explicit documentation of the number of excluded studies and reasons for exclusion at each stage.

### 2.1 Data sources and search strategy

A comprehensive literature search was performed across PubMed, Embase, Medline, the Cochrane Library, and Elsevier's ScienceDirect databases. Non-English language publications were excluded from the analysis. The search strategy incorporated relevant keywords and medical subject headings (MeSH) terms, including: ((persistent atrial fibrillation) OR (AF)) AND ((Pulsed field ablation) OR (PFA) OR (lack electroporation) OR (Farapulse)) AND ((radiofrequency ablation) OR (RFA) OR (cryoballoon ablation) OR (CBA) OR (traditional)). The literature search was last updated in March 2026.

### 2.2 Studies selection

Two reviewers (L-H and T-WP) independently performed the study screening and selection process. The inclusion criteria were as follows: (a) patients diagnosed with drug-refractory symptomatic PerAF who underwent ablation; (b) Patients undergoing their first CA procedure; (c) Comparative studies between PFA and either RFA or CBA; (d) Studies with a sample size of at least 20 participants; (e) Studies that provided comprehensive and reliable data on procedural outcomes, complications, and follow-up for both intervention groups. Excluded from the analysis were studies with unclear study designs or group allocations, as well as conference abstracts, case reports, case series, editorials, review articles, and publications in non-English languages.

### 2.3 Quality assessment and data extraction

Investigators (Q-QH, C-CF and C-Q) assessed the study quality using the Delphi consensus criteria for randomized controlled trials (RCTs) and the Newcastle-Ottawa Quality Assessment Scale (NOS) for observational research. The NOS comprises eight items, with a maximum score of nine points, utilizing a star system to evaluate study populations, group comparability, and the exposure/outcome of interest. Studies scoring ≥7 on the NOS were considered high quality (14). This systematic review adhered to the Preferred Reporting Items for Systematic Reviews and Meta-Analyses (PRISMA) guidelines and Cochrane Collaboration's recommendations. Risk of bias for the included non-randomized studies was comprehensively assessed using the ROBINS-I tool across seven predefined domains: confounding bias, selection bias, bias in classification of interventions, deviation from interventions, missing data bias, outcome measurement bias, and selection of reported results, with bias risk plots generated using the robvis software (15). Two reviewers extracted data collaboratively, resolving discrepancies through discussion to ensure consistency (16, 17).

The GRADE system is a standardized, transparent tool utilized to judge the overall certainty of clinical evidence. It offers a rigorous methodology for synthesizing evidence and formulating evidence-based clinical recommendations.This system considers five potential factors that may downgrade evidence certainty, including limi-tations in study design or execution, inconsistency in results, indirect-ness of evidence, imprecision of results, and publication bias. Additionally, three specific criteria are available to upgrade the evidence quality: magnitude of the effect, plausible residual confounding, and dose-response gradient. Following comprehensive assessment across all domains, the evidence certainty is categorized into four grades: high, moderate, low, and very low (18, 19). All evidence grading procedures in the present study were conducted via the online GRADEpro GDT platform (https://www.gradepro.org).

### 2.4 Outcome definitions

Procedure time: Defined as the duration from the administration of local anesthesia to the removal of all catheters.

Fluoroscopy time: Refers to the total duration of fluoroscopy from the procedure initiation to its completion.

Left atrial dwell (LAD) time: Denotes the period during which the catheter remains within the left atrium.

Complications: Included all-cause mortality, pericardial tamponade, persisting PNI, stroke/transient ischemic attack (TIA), esophageal lesions, and systemic thromboembolism.

Atrial tachyarrhythmias (ATAs) Recurrence: Defined as the any symptomatic or asymptomatic atrial arrhythmia (include AF, atrial tachycardia (AT) and atrial flutter(AFL)) lasting >30 s after the post-CA blanking period.

### 2.5 Statistical analysis

Two independent statisticians (L-H and C-CF) performed the meta-analysis by pooling summary statistics from individual trials. For dichotomous outcomes, risk ratios (RR) with 95% confidence intervals (CIs) were calculated, while continuous variables were analyzed using standard mean differences (SMD). Statistical analyses employed fixed and random-effects models, with weighting based on inverse variance methods following DerSimonian and Laird's approach (20). Between-study heterogeneity was evaluated using the *I^2^* statistic, where an *I^2^* > 50% indicated substantial heterogeneity. A *P* < 0.05 was considered statistically significant for all tests. We performed a leave-one-out sensitivity analysis to address heterogeneity by sequentially excluding each study from the pooled analysis, and If significant heterogeneity persisted, the random-effects model was applied. Statistical analyses were performed using Review Manager version 5.4 (The Nordic Cochrane Center, The Cochrane Collaboration, 2014, Copenhagen, Denmark) (21).

Publication bias was evaluated using funnel plots for outcomes. Egger's regression test was performed to formally assess asymmetry. No obvious asymmetry was observed on visual inspection of funnel plots, and Egger's test indicated no significant publication bias (all *P* > 0.05), suggesting that the pooled estimates were reliable.

## 3 Results

### 3.1 Eligible studies

The detailed literature screening process is depicted in Figure 1. An initial search retrieved 225 potentially relevant manuscripts, of which 12 were duplicate studies and 139 were excluded after title/abstract screening. Of the 74 articles selected for full-text review, 28 systematic reviews, 7 editorials/letters, and 8 case reports/series were excluded. Further evaluation of full texts led to exclusion of 25 studies due to flawed clinical design (7 studies), missing study endpoints (13 studies), and duplicate data reporting (5 studies). Ultimately, 6 studies were included in the analysis (22–27). The detailed assessment results of the Newcastle-Ottawa Scale (NOS) are presented in Table 1.

**Table 1:** Quality of evidence (in the included systematic reviews and meta-analysis assessed by GRADE.

Study	Risk of bias	Inconsistency	Indirectness	Imprecision	Publication Bias	Therapy Group	Control Group	Relative (95% CI)	Quality of evidence
Ahmed (27) (Procedure time)	Not serious	Serious^a^	Not serious	Not serious	Not serious	106 ± 34.1	143.5 ± 52.2	SMD −0.85 (−1.14 to −0.56)	㊉㊉㊉○ Moderate
Bianchini (22) (Procedure time)	Not serious	Serious^a^	Not serious	Not serious	Not serious	107.5 ± 28	166 48.5	SMD −1.45 (−1.90 to −0.99)	㊉㊉㊉○ Moderate
Isenegger (23) (Procedure time)	Not serious	Serious^a^	Not serious	Serious^b^	Not serious	49 ± 16.4	60. ± 19.4	SMD −0.61 (−0.88 to −0.34)	㊉㊉○○ Low
Kueffer (24) (Procedure time)	Not serious	Serious^a^	Not serious	Not serious	Not serious	60 ± 20.1	80 ± 24.6	SMD −0.89 (−1.10 to −0.69)	㊉㊉㊉○ Moderate
Pannone (25) (Procedure time)	Not serious	Serious^a^	Not serious	Not serious	Not serious	91.2 ± 19.5	108.4 ± 38.2	SMD −0.56 (−0.88 to −0.25)	㊉㊉㊉○ Moderate
Thomas (26) (Procedure time)	Serious^c^	Serious^a^	Not serious	Not serious	Not serious	60 ± 20	84 ± 24.4	SMD −1.08 (−1.29 to −0.87)	㊉㊉○○ Low
Ahmed (27) (Fluoroscopy time)	Not serious	Serious^a^	Not serious	Not serious	Not serious	19.5 ± 5.9	10 ± .5 7	SMD 1.39 (1.08 to 1.69)	㊉㊉㊉○ Moderate
Isenegger (23) (Fluoroscopy time)	Not serious	Serious^a^	Not serious	Not serious	Not serious	9 ± 3.7	11 ± 6	SMD −0.4 (−1.29 to −0.87)	㊉㊉㊉○ Moderate
Kueffer (24) (Fluoroscopy time)	Not serious	Serious^a^	Not serious	Not serious	Not serious	23 ± 9.25	19.7 ± 6.72	SMD 0.4 (0.21 to 0.6)	㊉㊉㊉○ Moderate
Pannone (25) (Fluoroscopy time)	Not serious	Serious^a^	Not serious	Not serious	Not serious	15.9 ± 3.5	30.1 ± 10.9	SMD −1.08 (−1.29 to −0.87)	㊉㊉㊉○ Moderate
Thomas (26) (Fluoroscopy time)	Serious^c^	Serious^a^	Not serious	Not serious	Not serious	23 ± 9.2	19.7 ± 6.7	SMD −1.08 (−1.29 to −0.87)	㊉㊉○○ Low
Ahmed (27) (LAD time)	Not serious	Serious^a^	Not serious	Not serious	Not serious	62 ± 23.7	98 ± 77	SMD −0.63 (−0.91 to −0.35)	㊉㊉㊉㊉ High
Bianchini (22) (LAD time)	Not serious	Serious^a^	Not serious	Not serious	Not serious	38 ± 14.1	37 ± 12.6	SMD 0.07 (−0.33 to 0.48)	㊉㊉㊉㊉ High
Isenegger (23) (LAD time)	Not serious	Serious^a^	Not serious	Not serious	Not serious	33 ± 13.4	37 ± 16.4	SMD −0.27 (−0.53 to −0.00)	㊉㊉㊉㊉ High
Pannone (25) (LAD time)	Not serious	Serious^a^	Not serious	Not serious	Not serious	60.5 ± 15.8	85.1 ± 24.5	SMD −1.19 (−1.52 to −0.85)	㊉㊉㊉○ Moderate
Ahmed (27) (ATAs)	Not serious	Not serious	Not serious	Not serious	Not serious	30/100	46/100	RR 0.65 (0.45 to 0.94)	㊉㊉㊉㊉ High
Bianchini (22) (ATAs)	Not serious	Not serious	Not serious	Not serious	Not serious	18/44	18/49	RR 1.11 (0.67 to 1.86)	㊉㊉㊉㊉ High
Isenegger (23) (ATAs)	Serious^d^	Not serious	Not serious	Not serious	Not serious	28/113	42/107	RR 0.63 (0.42 to 0.94)	㊉㊉㊉○ Moderate
Kueffer (24) (ATAs)	Not serious	Not serious	Not serious	Not serious	Not serious	80/214	70/190	RR 1.01 (0.79 to 1.31)	㊉㊉㊉㊉ High
Pannone (25) (ATAs)	Not serious	Not serious	Not serious	Serious^b^	Not serious	17/80	19/80	RR 0.89 (0.50 to 1.59)	㊉㊉㊉○ Moderate
Thomas (26) (ATAs)	Serious^c^	Not serious	Not serious	Not serious	Not serious	76/214	72/190	RR 0.94 (0.73 to 1.21)	㊉㊉㊉○ Moderate
Isenegger (23) (AF recurrence)	Not serious	Not serious	Not serious	Not serious	Not serious	24/113	35/107	RR 0.65 (0.42 to 1.02)	㊉㊉㊉㊉ High
Kueffer (24) (AF recurrence)	Not serious	Not serious	Not serious	Not serious	Not serious	62/214	63/190	RR 0.87 (0.65 to 1.17)	㊉㊉㊉㊉ High
Pannone (25) (AF recurrence)	Not serious	Not serious	Not serious	Serious	Not serious	9/80	13/80	RR 0.69 (0.31 to 1.53)	㊉㊉㊉○ Moderate
Isenegger (23) (AT/AFL recurrence)	Not serious	Not serious	Not serious	Not serious	Not serious	2/113	8/107	RR 0.24 (0.05 to 1.09)	㊉㊉㊉㊉ High
Kueffer (24) (AT/AFL recurrence)	Not serious	Serious^e^	Not serious	Not serious	Not serious	18/214	7/190	RR 2.28 (0.97 to 5.35)	㊉㊉㊉○ Moderate
Pannone (25) (AT/AFL recurrence)	Serious^d^	Not serious	Not serious	Not serious	Not serious	8/80	6/80	RR 1.33 (0.48 to 3.67)	㊉㊉㊉○ Moderate
Ahmed (27) (Complications)	Not serious	Not serious	Not serious	Not serious	Not serious	1/100	2/100	RR 0.50 (0.05 to 5.43)	㊉㊉㊉㊉ High
Bianchini (22) (Complications)	Not serious	Not serious	Not serious	Not serious	Not serious	0/44	6/49	RR 0.90 (0.00 to 1.47)	㊉㊉㊉㊉ High
Isenegger (23) (Complications)	Not serious	Not serious	Not serious	Not serious	Not serious	3/113	2/107	RR 1.42 (0.24 to 8.33)	㊉㊉㊉㊉ High
Kueffer (24) (Complications)	Not serious	Not serious	Not serious	Not serious	Not serious	5/214	5/190	RR 0.89 (0.26 to 3.02)	㊉㊉㊉㊉ High
Pannone (25) (Complications)	Not serious	Not serious	Not serious	Not serious	Not serious	0/80	1/80	RR 0.33 (0.01 to 8.06)	㊉㊉㊉㊉ High
Thomas (26) (Complications)	Serious^d^	Not serious	Not serious	Not serious	Not serious	5/214	0/190	RR 9.77 (0.54 to 75.57)	㊉㊉㊉○ Moderate

LAD, left atrial dwell; ATAs, atrial tachyarrhythmias; AF, atrial fibrillation; AT, atrial tachycardia; AFL, atrial flutter; SMD, standard mean difference; CI, conffdence interval; RR, risk ratio.

a High heterogeneity.

b 95% conffdence interval crosses 1.

c The authors did not perform a sensitive analysis by removing studies with a high risk of bias. The authors of the meta-analysis did not assess the risk of bias in the included studies and there is no description of the statistical weight of each study in the overall outcome.

d The authors did not perform a sensitive analysis by removing studies with a high risk of bias, but the statistical weight of studies with a high risk of bias contributes more than 50% to the overall outcome.

e Low similarity of point estimates and overlapping of their conffdence intervals.

### 3.2 Study characteristics

Table 2 summarizes the characteristics of the 6 included trials, involving a total of 1,610 patients (765 in the PFA group and 845 in the TA group).Most trials performed patients matching between groups for age, gender, body mass index (BMI), and left atrium diameter. All included studies were assessed to have good methodological quality, supporting the validity of this meta-analysis.

**Table 2:** Baseline characteristics of included study.

Study (year)	Country	Groups	Patients, (n)	Months since AF diagnosis, (Months)	Age, (years)	Male, (%)	BMI, (kg/m2)	Hy, (%)	DM, (%)	Stroke/TIA,(%)	CAD, (%)	LVEF, (%)	LAD, (mm)	Follow-up, (Months)	Design
Bianchini (22)	Italy	PFA	44	38.0 (18.5–102)	66.0 ± 7.4	79.6	27.2[24.0–31.2]	47.5	6.8	9.1	15.9	54.3 ± 11.6	45.5[36.5–57.5]^#^	12	Observational
		RFA	49	60.0 (46–120)	63.8 ± 10.6	81.6	27.1[24.3–31.0]	52.5	0	12.5	14.3	57.1 ± 9.9	45[38–57]^#^		
Kueffer (24)	Switzerland	PFA	214	12.0 ± 5.6	69.0[61–74]	76.6	28.1[24.9;32.3]	60.7	18.2	5.6	20.6	55.0[45.0;60.0]	45.0[38.0–53.0]^#^	12	Prospective Non-randomized
		RFA	129	9.0 ± 7.0	68.0[59–73]	69.8	28.8[25.2;32.2]	59.7	19.4	7.0	18.6	55.0[44.5;60.0]	44.0[36.8–50.8]^#^		
		CBA	190	20.0 ± 10.5	68.0[58–74]	72.6	29.0[25.1;32.7]	67.9	11.1	10.5	16.8	55.0[45.0;60.0]	40.0[31.2–50.8]^#^		
Pannone (25)	Belgium	PFA	80	10.4 ± 8.9	68.1 ± 8.6	72.5	NR	70.0	15	1.2	13.8	55.1 ± 8.2	46.2 ± 6.6	12	Retrospective
		CBA	80	11.2 ± 10.0	67.1 ± 9.0	67.5	NR	73.8	21.2	1,2	13.8	54.0 ± 7.9	45.5 ± 12.0		
Thomas (26)	USA	PFA	214	NR	69.0[61–74]	76.6	28.1[24.9–32.3]	60.7	NR	NR	20.6	NR	NR	12	Prospective registry
		CBA	190		68.2[58–74]	72.6	29.0[25.1–32.7]	67.9			16.8				
Isenegger (23)	Switzerland	PFA	113	NR	65.0[59–73]	78.0	27[25–31]	59.0	10.0	NR	11.0	54[45–59]	43[39–46]	12	Observational
		CBA	107		67.0[60–71]	74.0	28[25–31]	65,0	8.0		11.0	56[50–61]	42[39–47]		
Ahmed (27)	UK	PFA	100	16[10–30]	68[62.5–73]	72	30 ± 5.7	NR	NR	NR	NR	43.5[35.5–55.5]	48.5[42.8–55.5]	12.2 ± 2.8	Retrospective
		RFA	100	18[13–32]	68[60.5–76]	70	29.6 ± 5.1					47[40–58]	45.5[39.1–55]	12.2 ± 4.0	

Values are reported as the mean ± SD, medians (interquartile range), or *n* (%). PAF, paroxysmal atrial fibrillation; BMI, body mass index; CAD, coronary artery disease; DM, Diabetes mellitus; TIA, Transient ischemic attack; Hy, hypertension; LAD, left atrium diameter; NR, not recorded; PFA, pulsed field ablation; CBA, cryoballoon ablation; RFA, radiofrequency ablation; TA, Thermal ablation.

vHPRF.

LVEF < 35%.

# Left atrial volume index (mL/m2).

### 3.3 Quality assessment

After analyzing the studies using the Robins-I tool, assessments revealed varying degrees of bias across different domains.

Overall, one study was judged to have moderate risk of bias, mainly due to potential residual confounding, mild selection bias in participant enrollment, and a small amount of missing follow-up data (26). The remaining five studies were rated as low risk of bias in all domains, with adequate adjustment for confounders, clear definition of interventions, complete outcome data, and standardized outcome assessment (22–25, 27).These findings indicate that while some studies have potential flaws in confounding control and data completeness, others maintain high methodological rigor, ensuring robust research quality. RoB results are shown in Supplementary Figure S1.

We further evaluated the potential impact of bias on the pooled effect sizes. Studies with moderate risk were limited and did not dominate the pooled estimates. Sensitivity analyses excluding the study with moderate risk yielded similar results for atrial tachyarrhythmia recurrence, procedure time, and complication rates, suggesting that the overall findings remained robust and were not materially affected by bias.

The GRADE system was used to assess the quality of evidence across the following endpoints: procedural time, fluoroscopy time, LAD duration, ATAs, AF recurrence, AT/Aflu and complications. The final-GRADE analysis revealed 33 results, of which 14 results were of high quality, and 19 of moderate certainty. Full details of the GRADE ratings are listed in Supplementary Table S1.

### 3.4 Procedure outcomes

**Figure 2:**
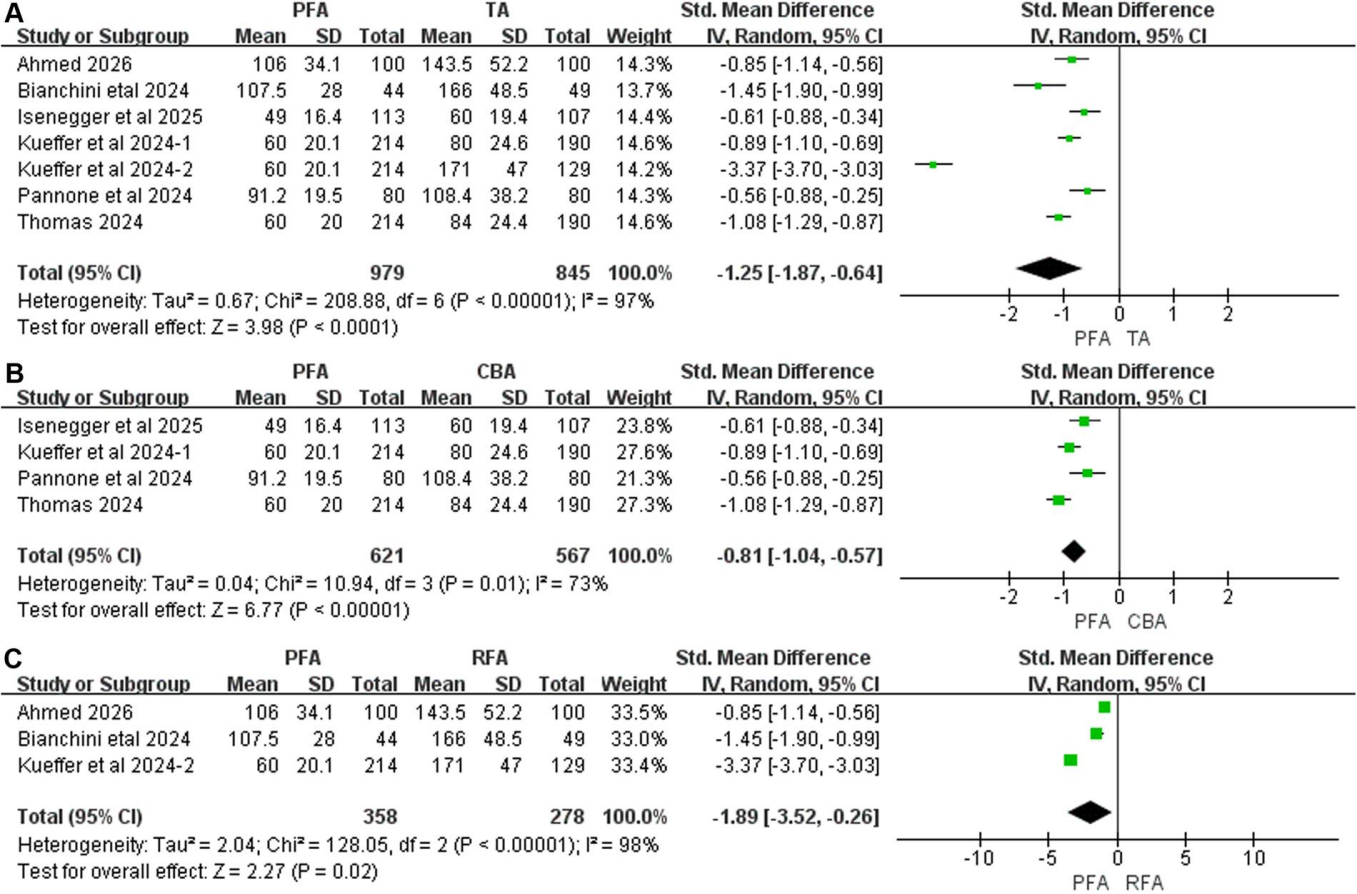
Forest plots comparing PFA and TA for procedure time. **(A)** Comparing PFA and TA; **(B)** Comparing PFA and CBA; **(C)** Comparing PFA and RFA. CI: confidence interval; SMD: standard mean difference; PFA: pulsed field ablation; TA: traditional ablation; CBA: cryoballoon ablation; RFA:radiofrequency ablation; LAD time: left atrial dwell time; *−*1: Trials comparing between PAF and CBA; *−*2:Trials comparing between PAF and RFA.

**Figure 3:**
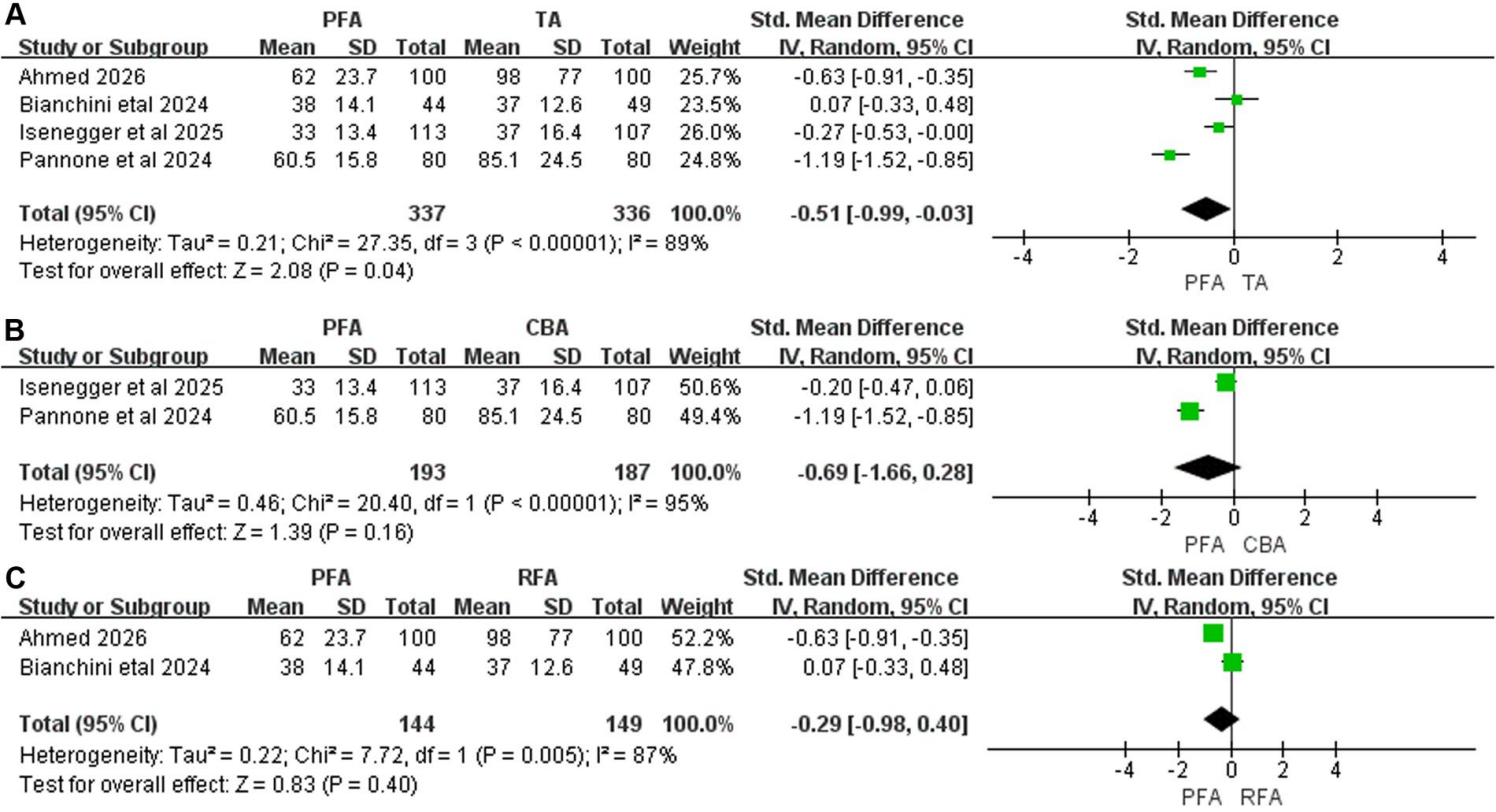
Forest plots comparing PFA and TA for LAD time. **(A)** Comparing PFA and TA; **(B)** Comparing PFA and CBA; **(C)** Comparing PFA and RFA. CI: confidence interval; SMD: standard mean difference; PFA: pulsed field ablation; TA: traditional ablation; CBA: cryoballoon ablation; RFA:radiofrequency ablation; LAD time: left atrial dwell time; *−*1: Trials comparing between PAF and CBA; −2:Trials comparing between PAF and RFA.

**Figure 4:**
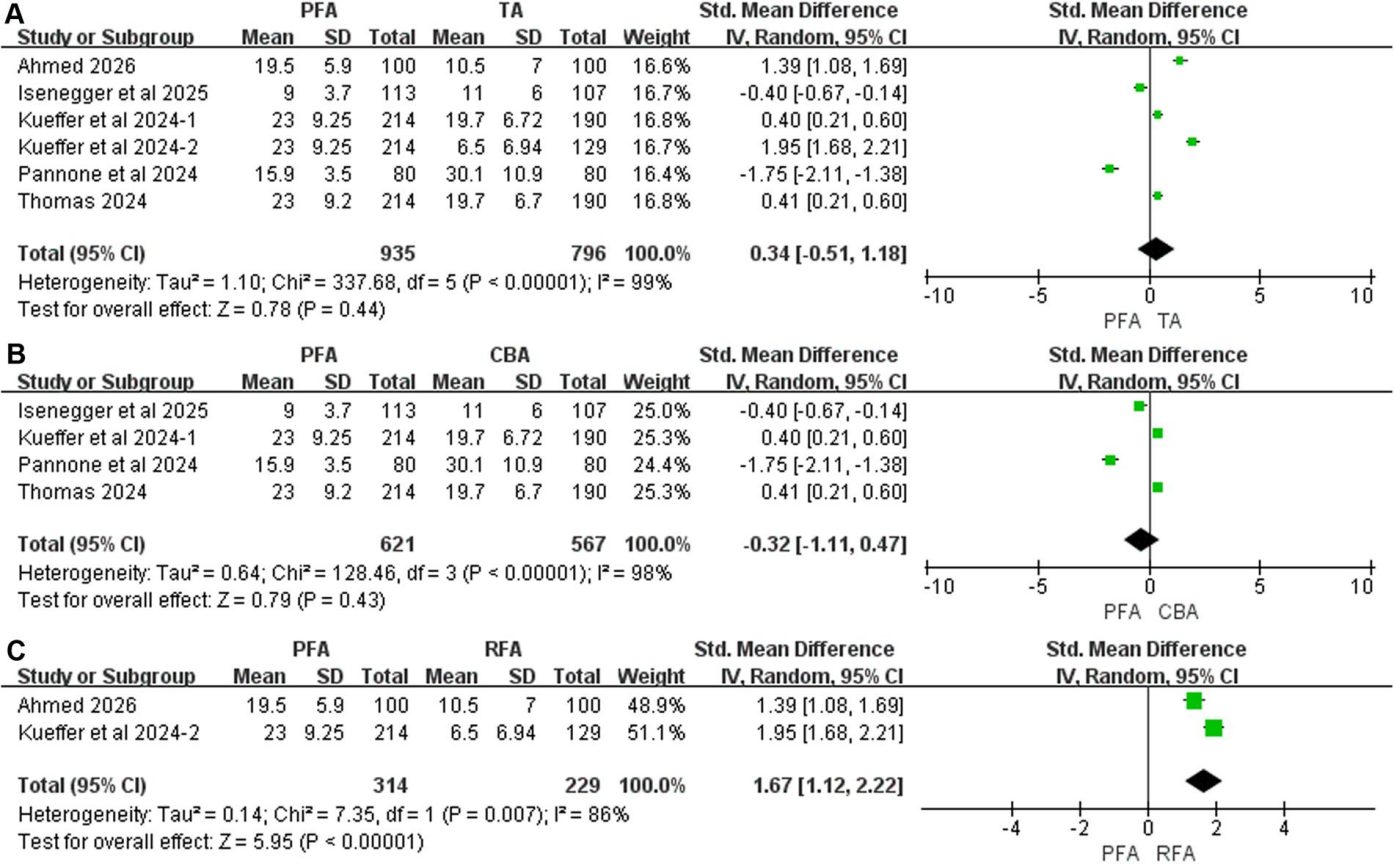
Forest plots comparing PFA and TA for fluoroscopy time. **(A)** Comparing PFA and TA; **(B)** Comparing PFA and CBA; **(C)** Comparing PFA and RFA. CI: confidence interval; SMD: standard mean difference; PFA: pulsed field ablation; TA: traditional ablation; CBA: cryoballoon ablation; RFA:radiofrequency ablation; LAD time: left atrial dwell time; −1: Trials comparing between PAF and CBA; *−*2: Trials comparing between PAF and RFA.

Pooled analysis demonstrated that PFA significantly shortened procedural time (SMD: −1.25, 95% CI: −1.87 to −0.64, *P* < 0.00001, Figure 2A) and LAD time (SMD: −0.51, 95% CI: −0.99 to −0.03, *P* = 0.04, Figure 3A). Further subgroup analyses in this study revealed that PFA conferred a significant advantage in procedural time when compared individually with either CBA or RFA (PFA vs. CBA, SMD: −0.81, 95% CI: −1.04 to −0.57, *P* < 0.00001 Figure 2B; PFA vs. RFA, SMD: −1.89, 95% CI: −3.52 to −0.26, *P* < 0.02 Figure 2C). For LAD time, when PFA was compared individually with CBA and RFA respectively, a trend toward reduction in LAD time was observed with PFA, without statistical significance(Figures 3B,C). In addition, PFA was not associated with a significant increase in fluoroscopy time relative to TA (SMD: 0.34, 95% CI: −0.51 to 1.18, *P* = 0.44 Figure 4A), when compared with CBA, identified the similar results (SMD: −0.32, 95% CI: −1.11 to 0.47, *P* = 0.43 Figure 4B) whereas PFA required a longer fluoroscopy time when compared individually with RFA (SMD: 1.67, 95% CI: 1.12 to 2.22, *P* < 0.00001 Figure 4C).

The studies included in the pooled analysis exhibited significant heterogeneity in procedural time (I^2^ = 97%), LAD time (I^2^ = 90%) and fluoroscopy time (I^2^ = 99%). Leave-one-out sensitivity analysis did not eliminate the heterogeneity. We therefore performed pre-specified subgroup analyses according to ablation technique (PFA vs. CBA; PFA vs. RFA) and meta-regression to explore potential sources of heterogeneity, including sample size, follow-up duration, operator experience, and proportion of long-standing persistent AF. Meta-regression indicated that ablation strategy and operator experience were significant contributors to between-study heterogeneity (all *P* < 0.05). Given the high heterogeneity, random-effects models were used for all analyses, and the overall direction and consistency of treatment effects remained stable.

Funnel plots were constructed to assess publication bias. Visual inspection of the plots for procedure time, fluoroscopy time and LAD time (Supplementary Figure S2A–C) revealed no significant asymmetry, suggesting no substantial publication bias.

### 3.5 Atrial tachyarrhythmias recurrence

**Figure 5:**
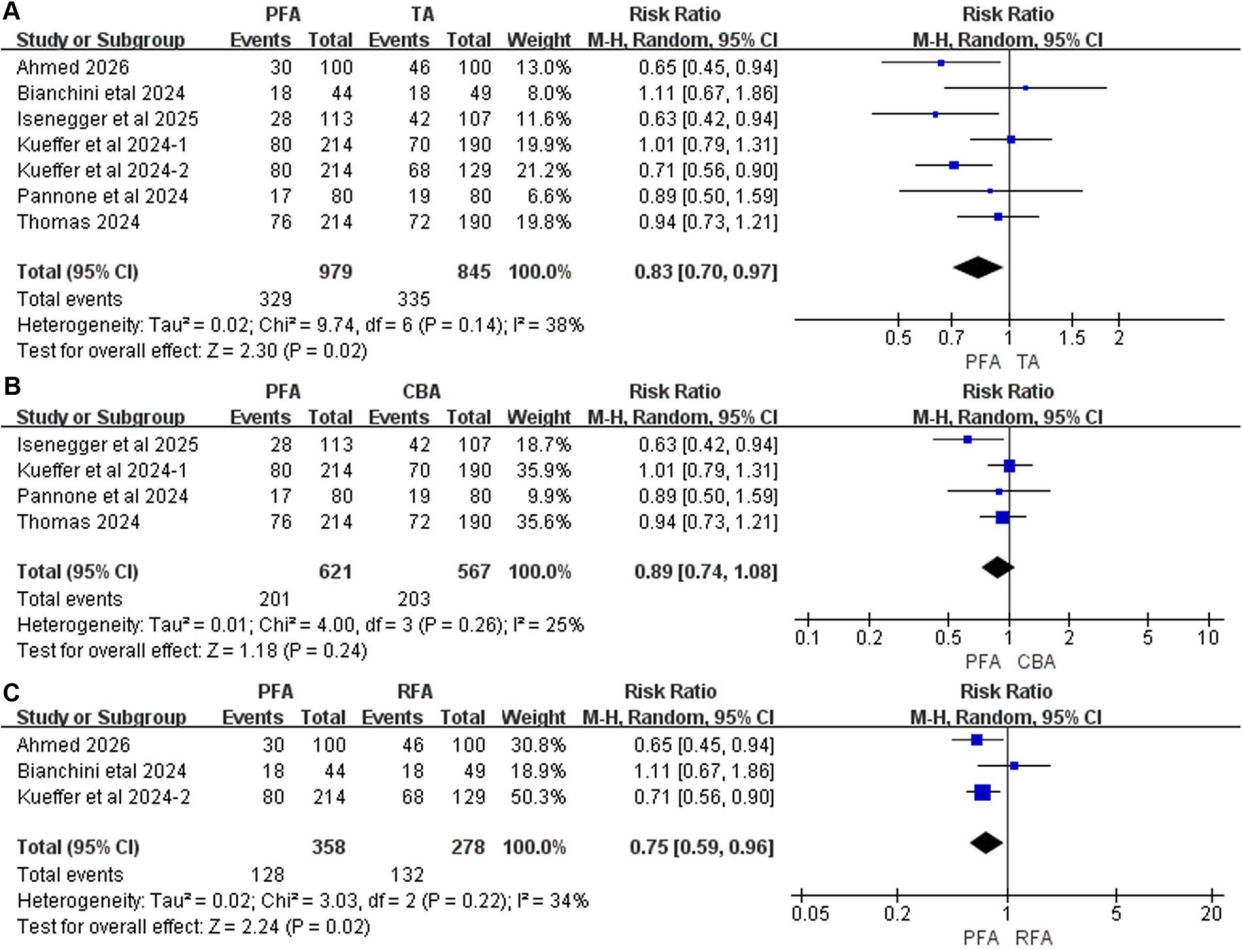
Forest plots comparing PFA and TA for ATAs recurrence. **(A)** Comparing PFA and TA; **(B)** Comparing PFA and CBA; **(C)** Comparing PFA and RFA. CI: confidence interval; RR: risk ratios; PFA: pulsed field ablation; TA: traditional ablation; CBA: cryoballoon ablation; RFA:radiofrequency ablation; ATAs: atrial tachyarrhythmias; *−*1: Trials comparing between PAF and CBA; *−*2: Trials comparing between PAF and RFA.

**Figure 6:**
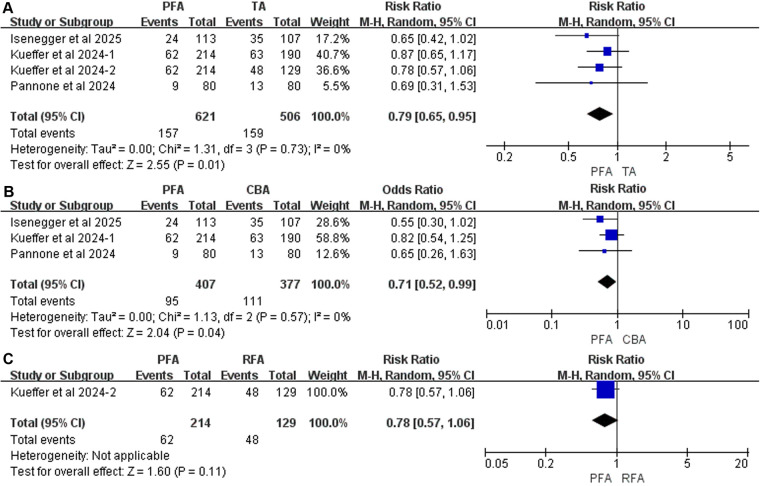
Forest plots comparing PFA and TA for AF recurrence. **(A)** Comparing PFA and TA; **(B)** Comparing PFA and CBA; **(C)** Comparing PFA and RFA. CI: confidence interval; RR: risk ratios; PFA: pulsed field ablation; TA: traditional ablation; CBA: cryoballoon ablation; RFA:radiofrequency ablation; AF: atrial fibrillation; *−*1: Trials comparing between PAF and CBA; *−*2: Trials comparing between PAF and RFA.

**Figure 7:**
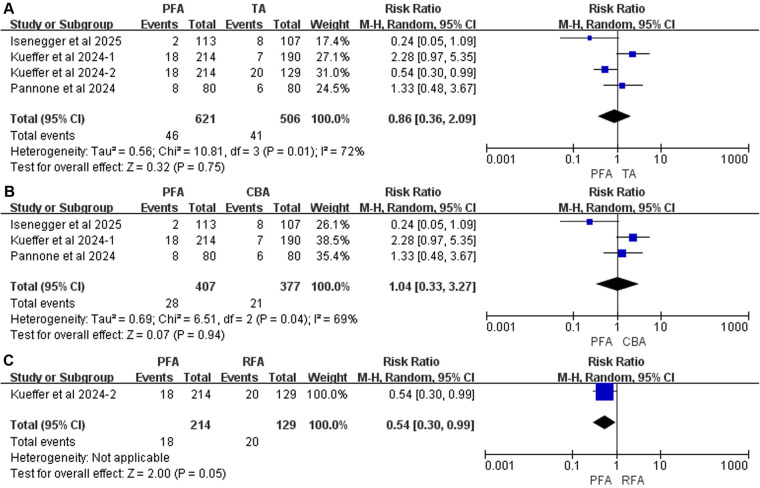
Forest plots comparing PFA and TA for AT/AFL recurrence. **(A)** Comparing PFA and TA; **(B)** Comparing PFA and CBA; **(C)** Comparing PFA and RFA. CI: confidence interval; RR: risk ratios; PFA: pulsed field ablation; TA: traditional ablation; CBA: cryoballoon ablation; RFA:radiofrequency ablation; AT/AFL: atrial tachycardia and atrial flutter; *−*1: Trials comparing between PAF and CBA; *−*2:Trials comparing between PAF and RFA.

PFA significantly reduced the risk of ATAs recurrence with low heterogeneity across studies (RR: 0.83, 95% CI: 0.70 to 0.97, *P* = 0.02, Figure 5A). This advantage was still observed when PFA was compared with RFA (RR: 0.75, 95% CI: 0.59 to 0.96, *P* = 0.02 Figure 5C), whereas statistical analysis indicated no significant difference between PFA and CBA (RR: 0.89, 95% CI: 0.74 to 1.08, *P* = 0.24 Figure 5B). PFA also conferred a significant advantage in reducing AF recurrence (RR: 0.79, 95% CI: 0.65 to 0.95, *P* = 0.015 Figure 6A). When compared individually with CBA, PFA still decreased AF recurrence (RR: 0.71, 95% CI: 0.52 to 0.99, *P* = 0.04 Figure 6B), while no statistical difference was found between RFA and PFA (RR: 0.78, 95% CI: 0.57 to 1.06, *P* = 0.11 Figure 6C). As for the recurrence of AT/AFL, no significant statistical difference was identified between the two groups (RR: 0.84, 95% CI: 0.64 to 1.04, *P* = 0.10, Figure 7A). Moderate heterogeneity was observed in the studies related to AT/AFL recurrence (I^2^ = 72%), and this heterogeneity persisted following leave-one-out sensitivity analysis.

Funnel plots were constructed to assess publication bias. Visual inspection of the plots for ATAs, AF recurrence and AT/AFL recurrence (Supplementary Figure S2D–F) revealed no significant asymmetry, suggesting no substantial publication bias.

### 3.6 Complications

**Figure 8:**
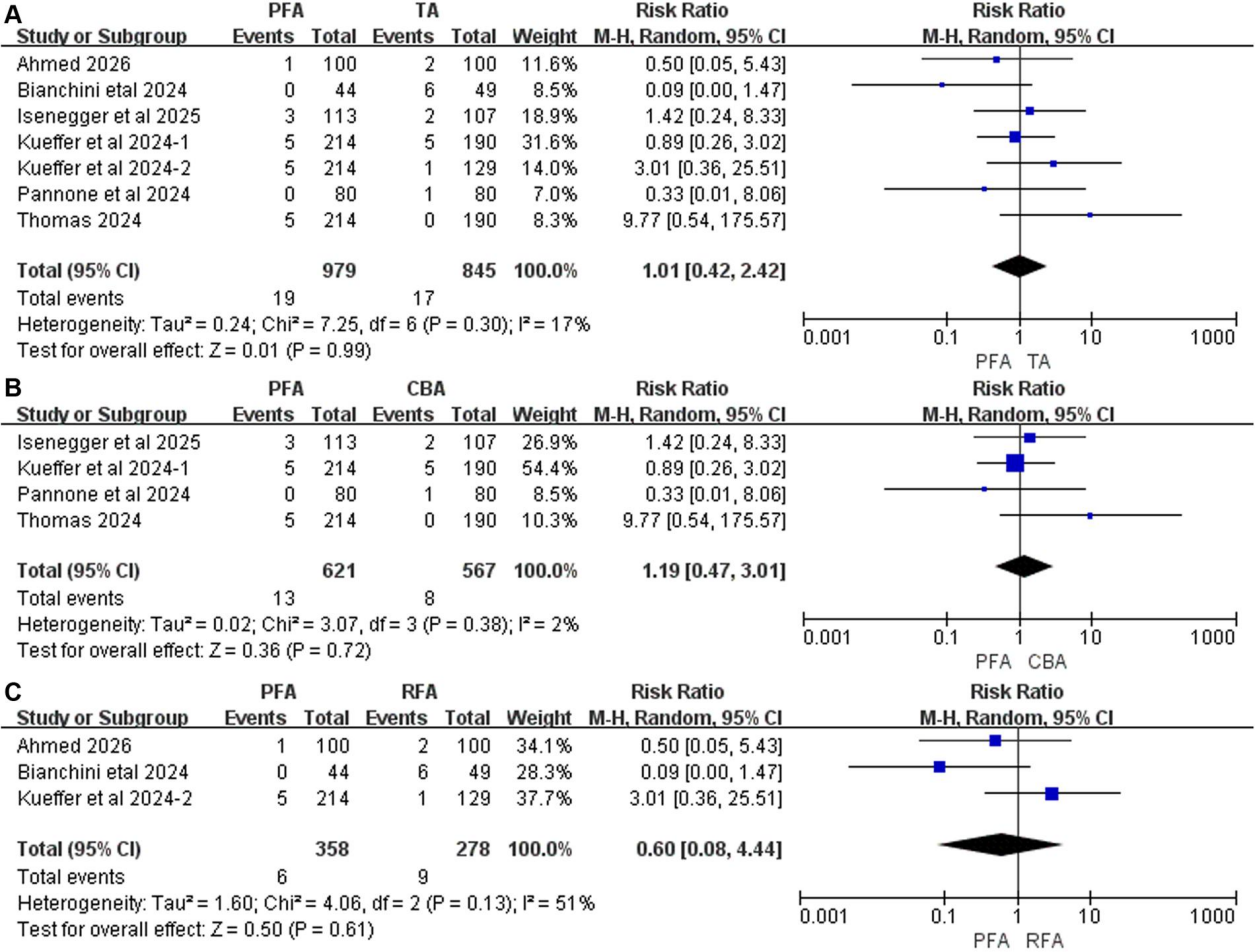
Forest plots comparing PFA and TA for complications. **(A)** Comparing PFA and TA; **(B)** Comparing PFA and CBA; **(C)** Comparing PFA and RFA. CI: confidence interval; RR: risk ratios; PFA: pulsed field ablation; TA: traditional ablation; CBA: cryoballoon ablation; RFA:radiofrequency ablation; *−*1: Trials comparing between PAF and CBA; *−*2:Trials comparing between PAF and RFA.

**Figure 9:**
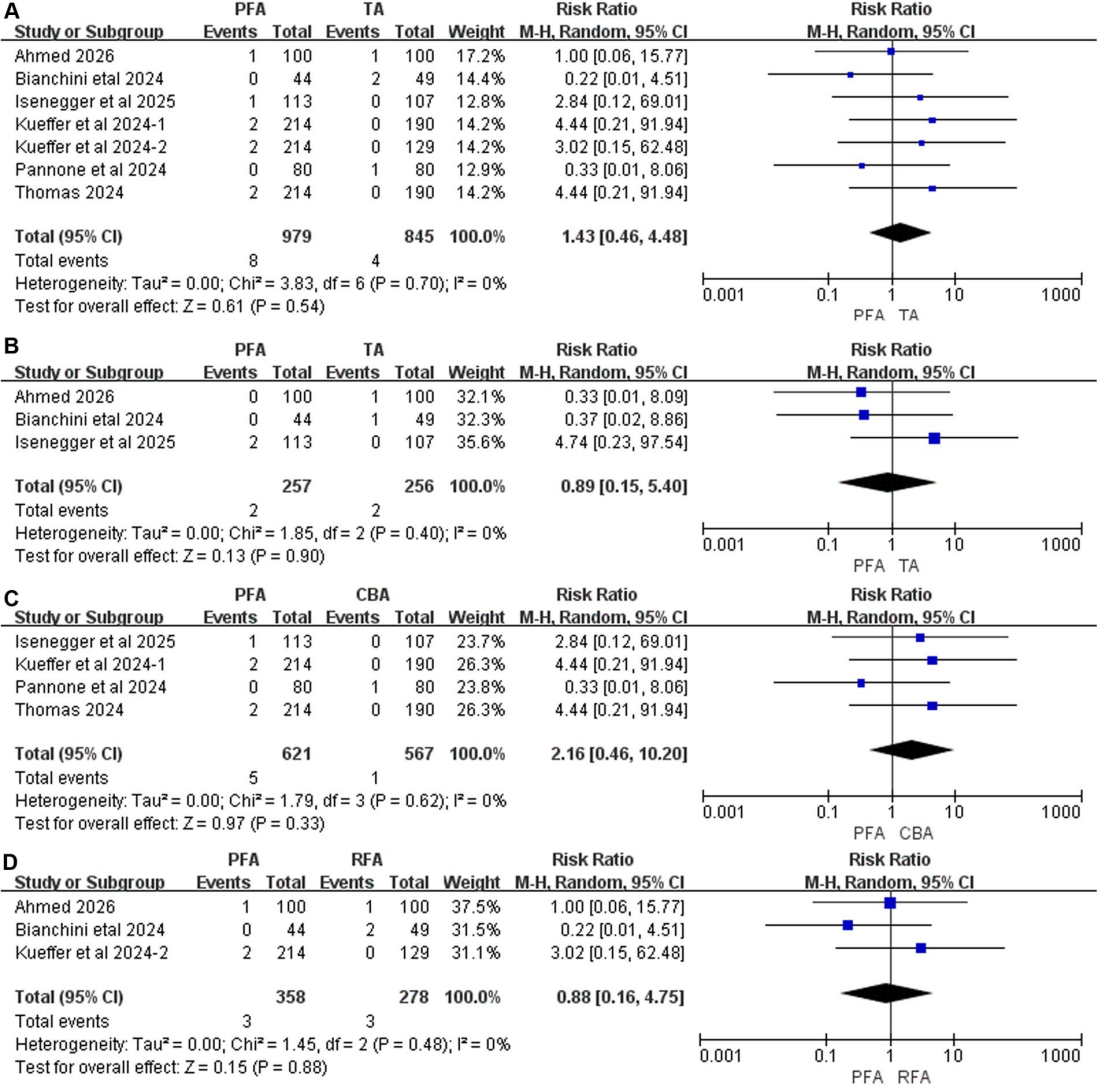
Forest plots comparing PFA and TA for cardiac tamponade and stroke. **(A)** Comparing PFA and TA for cardiac tamponade; **(B)** Comparing PFA and TA for stroke; **(C)** Comparing PFA and CBA for cardiac tamponade; **(D)** Comparing PFA and RFA for cardiac tamponade. CI: confidence interval; RR: risk ratios; PFA: pulsed field ablation; TA: traditional ablation; CBA: cryoballoon ablation; RFA:radiofrequency ablation; *−*1: Trials comparing between PAF and CBA; *−*2:Trials comparing between PAF and RFA.

There was no significant difference in the incidence of complications between the PFA and the TA (RR: 1.01, 95% CI: 0.42 to 2.42, *P* = 0.99, Figure 8A). PFA was not associated with an increased risk of complications when compared individually with either CBA or RFA (PFA vs. CBA: RR: 1.19, 95% CI: 0.47 to 3.01, *P* = 0.72 Figure 8B; PFA vs. RFA: RR: 0.60, 95% CI: 0.08 to 4.44, *P* = 0.61 Figure 8C). Similar results were observed between the two groups in terms of the incidence of cardiac tamponade and stroke (RR: 1.43, 95% CI: 0.46 to 4.48, *P* = 0.54, I^2^ = 0%, Figure 9A; RR: 0.89, 95% CI: 0.15 to 5.4, *P* = 0.40, I^2^ = 0%, Figure 9B). Further subgroup analysis of cardiac tamponade revealed no significant difference between the two groups in individual comparisons either (PFA vs. CBA: RR: 2.16, 95% CI: 0.46 to 10.20, *P* = 0.33 Figure 9C; PFA vs. RFA: RR: 0.88, 95% CI: 0.16 to 4.75, *P* = 0.61 Figure 9D). The studies included in the pooled analysis exhibited good homogeneity in the indicators related to complications.

Funnel plots were constructed to assess publication bias. Visual inspection of the plots for complications and cardiac tamponade (Supplementary Figures S2G, H) revealed no significant asymmetry, suggesting no substantial publication bias.

Among the 6 included studies, Bianchini L et al. adopted a hybrid convergent approach combining epicardial and endocardial radiofrequency ablation, which was distinctly different from the standard endocardial ablation used in other studies. To ensure methodological homogeneity and validate the robustness of the primary findings, we performed a sensitivity analysis excluding this study, with the remaining 5 studies included. The pooled effect sizes, direction, and statistical significance were fully consistent with the overall results (Supplementary Table S2; Supplementary Figure S3).

## 4 Discussion

This study represents an meta-analysis comparing the clinical outcomes using PFA vs. TA in patients with PerAF. The pooled analysis yields three key findings: (a) PFA significantly reduces the ATAs and AF recurrence; (b) PFA significantly shortens procedural time and LAD time; (c) overall complication show no significant difference between the two groups.

Our findings corroborate and extend with previous studies investing PVI in patients with PerAF with respect to recurrence-free survival and safety of PFA. PFA is a novel non-thermal technique that applies rapidly alternating high electric fields to induce nanopores in atrial tissue, causing cell death while preserving extracellular matrix integrity and reducing the risk of thermal damage to adjacent structures (9, 10). By selectively ablating cardiac tissue, PFA markedly decreases the risk of thermal injury to adjacent structures, establishing it as a versatile and safe modality for PVI (10). The MANIFEST-PF multicenter registry demonstrated that PFA was associated with no energy-related complications and shorter procedure time compared with TA (28). The ADVENT trial further indicated comparable complication rates between PFA and conventional thermal ablation (11).

Clinical evidence supporting PFA in PerAF remains limited. Kueffer et al. compared PFA with CBA and RFA in patients with PerAF. At 12-month follow-up, freedom from AF recurrence was 55% in the PFA group, 62% in the CBA group and 48% in RFA group, with improved arrhythmia-free survival observed in both the PFA and CBA groups compared to RFA (24). When excluding patients undergoing additional posterior wall isolation (PWI), the 1-year freedom from AF in the PFA group declined to 48%. Pannone et al. compared PFA and CBA for PVI combined with PWI, without three-dimensional mapping guidance and reported similar 1-year success rates (76.2% vs. 78.8%; *P* = 0.63) (25). Bianchini et al. found that ATAs recurrence at 1-year follow-up was non-inferior in the PFA group compared with mapping-guided RFA (36.7% vs. 40.9%, *P* = 0.68) (22).

Our findings are consistent with a recent meta-analysis by Mariani et al., which also demonstrated that PFA was associated with improved procedural efficiency and comparable safety compared with conventional thermal ablation in patients with atrial fibrillation (29). The present study extends these findings by specifically focusing on persistent AF and providing detailed quantitative comparisons of efficacy, procedural parameters, and long-term outcomes.

### 4.1 Procedure characteristics

This study's analysis reveals that, compared with TA, PFA significantly reduces procedure time and LAD time. The efficiency of PFA can be primarily attributed to its faster completion of individual pulmonary vein ablation and higher single-shot isolation rate, underscoring the technological advancements of single-shot devices in invasive treatment of AF (30, 31). Such rapid and efficient techniques hold promise for facilitating same-day discharge protocols following AF ablation procedures.

Non-fluoroscopic electroanatomical mapping systems are widely integrated in RFA, resulting in significantly shorter fluoroscopy time compared to CBA and PFA (11, 32–34). In PerAF, PFA tends to require longer fluoroscopic time, primarily due to PFA requires 4 to 8 ablations for pulmonary vein, each demanding x-ray visualization for precise catheter placement (32). However, operator experience has been shown to mitigate fluoroscopic exposure during PFA,and future integration of advanced mapping systems into PFA platforms may further curtail fluoroscopy reliance (34).

The pool analysis did reveal heterogeneity in procedure time, fluoroscopy time, and LAD time. Sensitivity analysis failed to identify a definite cause for this heterogeneity. We speculate that the heterogeneity might be attributed to differences in the proportion of PerAF across studies and variations in ablation strategies, which could contribute to the increased heterogeneity. Additionally, as multiple studies were from various global centers, differences in operators' ablation experience may also be contributing factors. To minimize the impact of heterogeneity, we adopted a random-effects model for statistical analysis, and the trends and consistency of the results were clear.

### 4.2 Efficiency

A synthesis of recent clinical evidence in this study confirmed that PFA achieved non-inferior efficacy to TA at 12-month follow-up, a finding validated by the MANIFEST-PF registry and the PULSED AF Pivotal Trial (35, 36). The ADVENT study reported similar results, showing a 73% recurrence-free rate in the PFA group vs. 71% in the TA group among patients with PAF (11). Currently, data on PerAF remain scarce. Additionally, several studies have indicated that PFA may induce early afterdepolarizations and ablation edge-related reentrant arrhythmias, with the incidence of new-onset AT/AFL ranging from 15% to 30% (24). However, the results of this meta-analysis demonstrated that PFA significantly reduced the risk of ATAs and AF recurrence, without increasing the risk of AT/AFL. When PFA was compared individually with RFA, PFA showed a trend toward reduced recurrence, albeit without statistical significance. This may be attributed to the fact that only one study was included for these two endpoints, and thus more research is required to validate these findings. Notably,it is critical to note that operators in these trials possessed expertise in TA but limited PFA experience (33). With the gradual improvement in operators' procedural proficiency and the integrated application of electroanatomical mapping and intracardiac echocardiography with PFA, the therapeutic index of PFA is expected to be further enhanced.

### 4.3 Safety

Although PFA exhibits a favorable initially safety profile, growing evidence has documented an increasing number of complications. Emerging studies have further demonstrated the diverse spectrum of PFA associated complications, including pericardial tamponade, stroke, coronary artery spasm, delayed ST-segment elevation, malignant arrhythmias, hemolysis-induced acute renal failure, and cardiac death ect (37, 38). The comprehensive analysis of included studies demonstrates that complication in both groups remained remarkably low and statistically indistinct, aligning with prior reports (11, 39). The diminished procedural risks are attributable to technological advancements, such as the use of contact force catheters and AI, as well as accumulated operator expertise (40, 41). Although operators performing PFA may have less experience, the technique's design inherently minimizes collateral tissue injury through selective targeting. In addition, compared with RFA, PFA has a shorter learning curve, with the optimization of procedures, the continuous accumulation of operators' experience, the update of new devices, the incidence of complications is expected to be further reduced (12, 39, 42).

## 5 Limitation

This meta-analysis has several limitations: 1). Publication Bias: As with any literature search, publication bias cannot be completely ruled out. Relying solely on published data may introduce bias; 2). Post-study registration may have introduced bias to our results and is a limitation of this study; 3)Study Design: More well-designed and larger RCTs are needed to validate these findings;4).Generalizability: Some studies had similar designs and ablation regimens, which may limit the generalizability of the results; 5).Follow-Up Strategies: Variations in follow-up strategies and tools across different studies increase potential heterogeneity. Overall, these limitations suggest the need for more comprehensive and standardized research to strengthen the conclusions drawn from this meta-analysis.

## 6 Conclusion

This meta-analysis suggests that PFA is a promising alternative to TA for PerAF, with potential benefits in reducing ATAs, shortening procedural time and LAD time, while showing comparable safety. These findings need to be further validated in large, RCTs with standardized lesion sets and uniform technology platforms.

## Data Availability

The original contributions presented in the study are included in the article/Supplementary Material, further inquiries can be directed to the corresponding author/s.
