# Supplementary material for: Meta-analysis of the safety and efficacy between pulsed field ablation vs. traditional ablation for persistent atrial fibrillation
Source: Front Cardiovasc Med. 2026 Aug 3;13:1839858. doi: 10.3389/fcvm.2026.1839858 (PMC13478584; doi:10.3389/fcvm.2026.1839858)
Supplement: Supplementary file 1 [file Supplementaryfile1.docx]

| **Table S1 Sensitivity analysis excluding the study by Bianchini et al.** | | | | | |
| --- | --- | --- | --- | --- | --- |
| **Outcome** | **Group** | **Pooled Effect** | **95% CI** | **P-value** | **I² (%)** |
| **Procedure time** | PFA vs TA | SMD=−1.22 | −1.91 to −0.54 | 0.0005 | 98 |
| **Fluoroscopy time** | PFA vs TA | SMD=0.34 | −0.51 to 1.18 | 0.44 | 99 |
| **Left atrial dwell time** | PFA vs TA | SMD=−0.66 | −1.12 to −0.12 | 0.02 | 90 |
| **ATAs** | PFA vs TA | RR=0.81 | 0.68–0.95 | 0.01 | 30 |
| **AF recurrence** | PFA vs TA | RR=0.79 | 0.65–0.95 | 0.01 | 0 |
| **AT/AFL recurrence** | PFA vs TA | RR=0.86 | 0.36–2.09 | 0.75 | 72 |
| **Complications** | PFA vs TA | RR=1.22 | 0.55–2.69 | 0.62 | 0 |
| **Cardiac tamponade** | PFA vs TA | RR=1.96 | 0.57–6.73 | 0.83 | 0 |
| PFA: pulsed field ablation; TA: traditional ablation; AF: atrial fibrillation; ATAs:Atrial tachyarrhythmias; AT: atrial tachycardia; AFL: atrial flutter; CI: Confidence Interval; SMD:Standardized Mean Difference; RR:Risk Ratio. | | | | | |
