# Supplementary material for: Meta-analysis of the safety and efficacy between pulsed field ablation vs. traditional ablation for persistent atrial fibrillation
Source: Front Cardiovasc Med. 2026 Aug 3;13:1839858. doi: 10.3389/fcvm.2026.1839858 (PMC13478584; doi:10.3389/fcvm.2026.1839858)
Supplement: Supplementary file 2 [file Table1.docx]

| Table S1. Quality of evidence ( in the included Systematic Reviews and Meta-analysis assessed by GRADE | | | | | | | | | |
| --- | --- | --- | --- | --- | --- | --- | --- | --- | --- |
| Study | Risk of  bias | Inconsistency | Indirectness | Imprecision | Publication  Bias | Therapy  Group | Control  Group | Relative (95 % CI) | Quality of evidence |
| Ahmed 2026  (Procedure time) | not serious | serious^a^ | not serious | not serious | not serious | 106±34.1 | 143.5±52.2 | SMD −0.85  (-1.14 to -0.56) | ㊉㊉㊉◯ Moderate |
| Bianchini 2024  (Procedure time) | not serious | serious^a^ | not serious | not serious | not serious | 107.5±28 | 166 48.5 | SMD −1.45  (-1.90 to -0.99) | ㊉㊉㊉◯ Moderate |
| Isenegger 2025  (Procedure time) | not serious | serious^a^ | not serious | serious^b^ | not serious | 49±16.4 | 60.±19.4 | SMD −0.61  (-0.88 to -0.34) | ㊉㊉◯◯ Low |
| Kueffer 2024  (Procedure time) | not serious | serious^a^ | not serious | not serious | not serious | 60±20.1 | 80±24.6 | SMD −0.89  (-1.10 to -0.69) | ㊉㊉㊉◯ Moderate |
| Pannone 2024  (Procedure time) | not serious | serious^a^ | not serious | not serious | not serious | 91.2±19.5 | 108.4±38.2 | SMD −0.56  (-0.88 to -0.25) | ㊉㊉㊉◯ Moderate |
| Thomas 2024  (Procedure time) | serious^c^ | serious^a^ | not serious | not serious | not serious | 60±20 | 84±24.4 | SMD −1.08  (-1.29 to -0.87) | ㊉㊉◯◯ Low |
| Ahmed 2026  (Fluoroscopy time) | not serious | serious^a^ | not serious | not serious | not serious | 19.5±5.9 | 10±.5 7 | SMD 1.39  (1.08 to 1.69) | ㊉㊉㊉◯ Moderate |
| Isenegger 2025  (Fluoroscopy time) | not serious | serious^a^ | not serious | not serious | not serious | 9±3.7 | 11±6 | SMD −0.4  (-1.29 to -0.87) | ㊉㊉㊉◯ Moderate |
| Kueffer 2024  (Fluoroscopy time) | not serious | serious^a^ | not serious | not serious | not serious | 23±9.25 | 19.7±6.72 | SMD 0.4  (0.21 to 0.6) | ㊉㊉㊉◯ Moderate |
| Pannone 2024  (Fluoroscopy time) | not serious | serious^a^ | not serious | not serious | not serious | 15.9±3.5 | 30.1±10.9 | SMD −1.08  (-1.29 to -0.87) | ㊉㊉㊉◯ Moderate |
| Thomas 2024  (Fluoroscopy time) | serious^c^ | serious^a^ | not serious | not serious | not serious | 23±9.2 | 19.7±6.7 | SMD −1.08  (-1.29 to -0.87) | ㊉㊉◯◯ Low |
| Ahmed 2026  (LAD time) | not serious | serious^a^ | not serious | not serious | not serious | 62±23.7 | 98±77 | SMD −0.63  (-0.91 to -0.35) | ㊉㊉㊉㊉ High |
| Bianchini 2024  (LAD time) | not serious | serious^a^ | not serious | not serious | not serious | 38±14.1 | 37±12.6 | SMD 0.07  (-0.33 to 0.48) | ㊉㊉㊉㊉ High |
| Isenegger 2025  (LAD time) | not serious | serious^a^ | not serious | not serious | not serious | 33±13.4 | 37±16.4 | SMD −0.27  (-0.53 to -0.00) | ㊉㊉㊉㊉ High |
| Pannone 2024  (LAD time) | not serious | serious^a^ | not serious | not serious | not serious | 60.5±15.8 | 85.1 ±24.5 | SMD −1.19  (-1.52 to -0.85) | ㊉㊉㊉◯ Moderate |
| Ahmed 2026  (ATAs) | not serious | not serious | not serious | not serious | not serious | 30/100 | 46/100 | RR 0.65 (0.45 to 0.94) | ㊉㊉㊉㊉ High |
| Bianchini 2024  (ATAs) | not serious | not serious | not serious | not serious | not serious | 18/44 | 18/49 | RR 1.11 (0.67 to 1.86) | ㊉㊉㊉㊉ High |
| Isenegger 2025  (ATAs) | serious^d^ | not serious | not serious | not serious | not serious | 28/113 | 42/107 | RR 0.63 (0.42 to 0.94) | ㊉㊉㊉◯ Moderate |
| Kueffer 2024  (ATAs) | not serious | not serious | not serious | not serious | not serious | 80/214 | 70/190 | RR 1.01 (0.79 to 1.31) | ㊉㊉㊉㊉ High |
| Pannone 2024  (ATAs) | not serious | not serious | not serious | serious^b^ | not serious | 17/80 | 19/80 | RR 0.89 (0.50 to 1.59) | ㊉㊉㊉◯ Moderate |
| Thomas 2024  (ATAs) | serious^c^ | not serious | not serious | not serious | not serious | 76/214 | 72/190 | RR 0.94 (0.73 to 1.21) | ㊉㊉㊉◯ Moderate |
| Isenegger 2025  (AF recurrence) | not serious | not serious | not serious | not serious | not serious | 24/113 | 35/107 | RR 0.65 (0.42 to 1.02) | ㊉㊉㊉㊉ High |
| Kueffer 2024  (AF recurrence) | not serious | not serious | not serious | not serious | not serious | 62/214 | 63/190 | RR 0.87 (0.65 to 1.17) | ㊉㊉㊉㊉ High |
| Pannone 2024  (AF recurrence) | not serious | not serious | not serious | serious | not serious | 9/80 | 13/80 | RR 0.69 (0.31 to 1.53) | ㊉㊉㊉◯ Moderate |
| Isenegger 2025  (AT/AFL recurrence) | not serious | not serious | not serious | not serious | not serious | 2/113 | 8/107 | RR 0.24 (0.05 to 1.09) | ㊉㊉㊉㊉ High |
| Kueffer 2024  (AT/AFL recurrence) | not serious | serious^e^ | not serious | not serious | not serious | 18/214 | 7/190 | RR 2.28 (0.97 to 5.35) | ㊉㊉㊉◯ Moderate |
| Pannone 2024  (AT/AFL recurrence) | serious^d^ | not serious | not serious | not serious | not serious | 8/80 | 6/80 | RR 1.33 (0.48 to 3.67) | ㊉㊉㊉◯ Moderate |
| Ahmed 2026  (Complications) | not serious | not serious | not serious | not serious | not serious | 1/100 | 2/100 | RR 0.50 (0.05 to 5.43) | ㊉㊉㊉㊉ High |
| Bianchini 2024  (Complications) | not serious | not serious | not serious | not serious | not serious | 0/44 | 6/49 | RR 0.90 (0.00 to 1.47) | ㊉㊉㊉㊉ High |
| Isenegger 2025  (Complications) | not serious | not serious | not serious | not serious | not serious | 3/113 | 2/107 | RR 1.42 (0.24 to 8.33) | ㊉㊉㊉㊉ High |
| Kueffer 2024  (Complications) | not serious | not serious | not serious | not serious | not serious | 5/214 | 5/190 | RR 0.89 (0.26 to 3.02) | ㊉㊉㊉㊉ High |
| Pannone 2024  (Complications) | not serious | not serious | not serious | not serious | not serious | 0/80 | 1/80 | RR 0.33 (0.01 to 8.06) | ㊉㊉㊉㊉ High |
| Thomas 2024  (Complications) | serious^d^ | not serious | not serious | not serious | not serious | 5/214 | 0/190 | RR 9.77 (0.54 to 75.57) | ㊉㊉㊉◯ Moderate |
| LAD: left atrial dwell; ATAs: atrial tachyarrhythmias; AF: atrial fibrillation; AT: atrial tachycardia; AFL: atrial flutter; SMD: standard mean difference; CI: conffdence interval; RR: risk ratio. Explanations: (a) High heterogeneity. (b) 95 % conffdence interval crosses 1. (c) The authors did not perform a sensitive analysis by removing studies with a high risk of bias. The authors of the meta-analysis did not assess the risk of bias in the included studies and there is no description of the statistical weight of each study in the overall outcome. (d) The authors did not perform a sensitive analysis by removing studies with a high risk of bias, but the statistical weight of studies with a high risk of bias contributes more than 50 % to the overall outcome. (e)Low similarity of point estimates and overlapping of their conffdence intervals. | | | | | | | | | |
